# Non-functional allele of *RESTORER OF FERTILITY 4* is functional for the reduction of *orf288* RNA in *japonica* rice

**DOI:** 10.5511/plantbiotechnology.25.1116a

**Published:** 2026-03-25

**Authors:** Kinya Toriyama, Yuko Iwai, Shinya Takeda, Ayumu Takatsuka, Keisuke Igarashi, Tomohiko Kazama

**Affiliations:** 1 Graduate School of Agricultural Science, Tohoku University, Sendai, Miyagi 980-8572, Japan; 2 Graduate School of Bioresource and Bioenvironmental Science, Kyushu University, Fukuoka, Fukuoka 819-0395, Japan

**Keywords:** cytoplasmic male sterility, mitochondrial gene, *Oryza glaberrima*, *Oryza sativa*, PPR gene

## Abstract

Cytoplasmic male sterility (CMS) is driven by the incompatibility between a mitochondrial gene and the nuclear *RESTORER OF FERTILITY* (*Rf*) gene. Previously, we identified *orf288* as the cryptic CMS-causing gene in the mitochondrial genome of *japonica* rice (*Oryza sativa*) Taichung 65 (T65) through the analysis of CMS lines of *O. glaberrima* carrying the cytoplasm of *japonica* rice; *orf288* RNA was greatly reduced in the presence of a T65-derived nuclear *Rf* gene, which was linked to a region harboring *rf4* encoding a pentatricopeptide repeat (PPR) protein and other *Rf*-like PPR genes. In this study, we knocked out *Rf-*like PPR genes in T65 to identify the *Rf*-like PPR gene regulating the reduction of *orf288* RNA and pollen fertility. Among the CRISPR-Cas9-mediated mutations in *PPR461* (Os10g0495100), *PPR782*/*rf4* (Os10g0495200), and *PPR794* (Os10g0497300), mutations in *PPR782*/*rf4* (Os10g0495200) allowed the expression of *orf288*; however, the *orf288* RNA level was lower than that in the *O. glaberrima* CMS line and did not affect pollen fertility. Our results indicate that *PPR782*/*rf4*, a non-functional allele of the *RF4* gene responsible for fertility restoration in wild-abortive (WA)-type CMS, is functional and suppresses the accumulation of *orf288* RNA in the *japonica* rice.

## Introduction

Cytoplasmic male sterility (CMS), associated with the dysfunctions in pollen or anther development, is an agronomically important trait used in F_1_ hybrid breeding. CMS plants are frequently generated by successive backcrossing between distantly related species, which triggers imbalanced interactions between the mitochondrial and nuclear genomes (Toriyama 2021, for a review).

Previously, we reported that an Asian *japonica* rice cultivar (*Oryza sativa* L.), ‘Taichung 65’ (T65) or ‘Nipponbare’ (NIP), exhibited CMS when the nucleus was replaced by African rice *O. glaberrima* Steud. (Toriyama et al. 2024). CMS lines produced irregular pollen grains and did not develop seeds. Fertility was restored by the action of a *RESTORER OF FERTILITY* (*Rf*) gene on chromosome 10, which was derived from T65 or NIP. The *Rf* gene is linked to a single sequence repeat marker, SSRH10045, in a region corresponding to some pentatricopeptide repeat (PPR)-containing genes cloned as *Rf* genes, such as *Rf1a* and *Rf1b* for Boro-Taichung 65 (BT)-CMS (Akagi et al. 2004; Kazama and Toriyama 2003; Komori et al. 2004; Wang et al. 2006), *Rf5* for Hong-Lian (HL)-CMS (Hu et al. 2012), *Rf4* for wild-abortive (WA)-CMS (Kazama and Toriyama 2014; Tang et al. 2014), *Rf98* for RT98-CMS (Igarashi et al. 2016), *OsRf19* for Fujian CMS (Jiang et al. 2022), and *Rfta* for Tadukan-CMS (Takatsuka et al. 2025). PPR proteins, in general, are involved in RNA processing including editing, cleavage, degradation, and blocking translation in mitochondria and plastids (Gaborieau et al. 2016; Kazama and Toriyama 2003; Schmitz-Linneweber and Small 2008; Wang et al. 2006). Considering the high expression of the mitochondrial *orf288* gene in the *O. glaberrima* CMS line with T65 cytoplasm (TGA) while the reduced expression in the restorer lines (TGR) or T65 (Toriyama et al. 2024), we hypothesized that the *Rf* gene associated with TGA would be an *Rf*-like PPR gene.

To confirm that *orf288* is a CMS-inducing gene, we produced *orf288-*depleted T65 plants using Mito-TALEN, a genome-editing tool for mitochondria (Toriyama et al. 2024). The *orf288*-depleted cytoplasm did not confer male sterility when backcrossed with *O. glaberrima*. Hence, *orf288* was considered a cryptic CMS-causing gene in mitochondrial genomes of *japonica* rice (Toriyama et al. 2024).

The *orf288* gene harbors a sequence similar to a part of the CMS-causing gene *WA352*, associated with WA-CMS (Luo et al. 2013; Tang et al. 2017; Zhou et al. 2024). The *Rf* gene for the WA-CMS derived from ‘IR 24’ is *PPR782a*/*Rf4* (Kazama and Toriyama 2014). *PPR782a*/*Rf4* encodes a pentatricopeptide repeat protein and functions in the reduction of the *WA352* RNA level, resulting in fertility restoration (Kazama and Toriyama 2014). A non-restorer line, NIP, contains a non-functional allele *of Rf4* (Locus ID, Os10g0495200; Rice Annotation Project Database [RAP-DB]) (Kawahara et al. 2013; Sakai et al. 2013), whose amino acid sequence shows 95% identity to that of *PPR782a*/*Rf4* (Kazama and Toriyama 2014).

Although *orf288* is not expressed in the anthers of NIP, the ORF288 protein expressed in yeast binds to COX11, a subunit of the cytochrome c oxidase complex, in the same manner as the WA352 protein (Tang et al. 2017). Therefore, we considered that knocking out certain *Rf*-like PPR genes in T65 cells may allow the expression of the mitochondrial *orf288* gene, inducing CMS via interaction with COX11, as reported in WA-CMS.

Hence, in this study, to identify the *Rf*-like PPR gene regulating the *orf288* RNA accumulation and pollen fertility, we have knocked out three *Rf*-like PPR genes in T65 using CRISPR-Cas9 and investigated the accumulation of *orf288* RNA and seed setting rates. Although CMS plants have not yet been obtained, our results indicate that the T65 allele of *PPR782a*/*rf4*, which is non-functional for reducing *WA352* RNA, is functional for reducing *orf288* RNA in *japonica* rice. A possible method to create CMS plants is discussed.

## Materials and methods

### Sequencing of *Rf*-like PPR genes

DNA was extracted from young leaves of T65 (*O. sativa*) using NucleoBond™ High Molecular Weight DNA Kit (Macherey-Nagel). HiFi reads (>10 kb) generated by the PacBio Sequel II sequencer were assembled under a 1-kb overlapping condition, following the method described by Takatsuka et al. (2025). The structure and number of PPR motifs in each PPR protein were predicted using PPRFinder (Cheng et al. 2016; Gutmann et al. 2020; https://ppr.plantenergy.uwa.edu.au/ (Accessed Mar 2, 2023)).

The *O. glaberrima* CMS (TGA) and restorer (TGR) lines were obtained using *O. glaberrima* (IRGC103777) as the maintainer line, as previously described (Toriyama et al. 2024). The total DNA of *O. glaberrima* was isolated using the DNeasy Plant Mini Kit (Qiagen). Individual PPR genes, corresponding to *PPR461*, *PPR782*/*rf4*, and *PPR794*, were amplified through PCR using the primers listed in Supplementary Table S1. Nucleotide sequences were determined by direct sequencing of PCR products using the primers listed in Supplementary Table S1.

### CRISPR-Cas9 knockout of PPR genes

**Figure 1:**
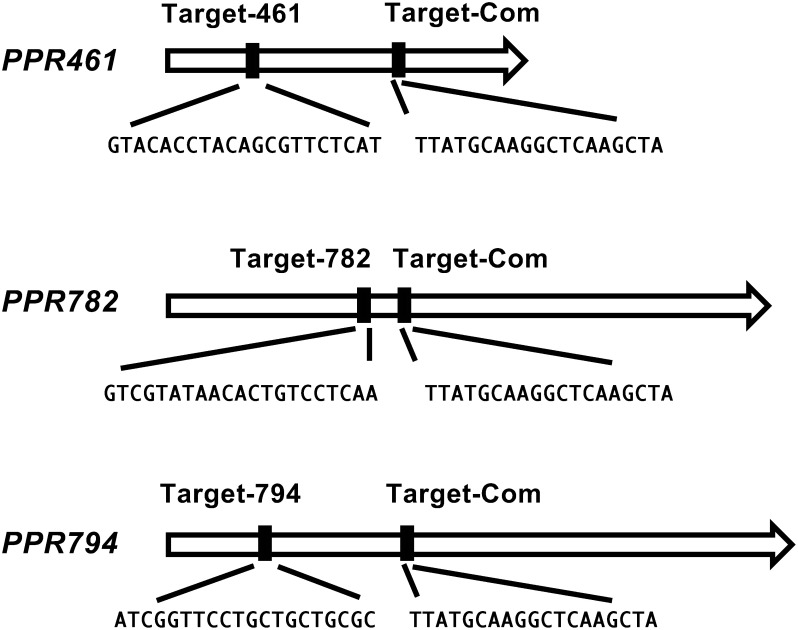
Figure 1. Common and gene-specific guide RNAs.

Guide RNAs were designed based on the common sequence (Target-Com) of *PPR461*, *PPR782*/*rf4*, and *PPR794*, and the gene-specific sequences (Target-461, Target-782, and Target-794) (Figure 1). The CRISPR-Cas9 vector derived from pZH_gYSA_MMCas9 (Mikami et al. 2015) was introduced into T65 and the *orf288*-depleted T65 (Toriyama et al. 2024) via *Agrobacterium*-mediated transformation (Suketomo et al. 2020). The presence of the introduced CRISPR-Cas9 vector was confirmed by detecting the hygromycin phosphotransferase (HPT) gene following the previously described method (Toriyama et al. 2024). Nucleotide sequences in and around the target sites were determined through direct sequencing of the PCR products using the primers listed in Supplementary Table S2. Chromatograms of each nucleotide sequence were analyzed using the ICE CRISPR Analysis Tool (https://ice.editco.bio/#/ (Accessed Jan 11, 2024)). HPT primers were used to select null segregants of the introduced T-DNA. The presence of *orf288* was confirmed through PCR, as described previously (Toriyama et al. 2024). Plants were grown in a biotron (LPH-410SPC; Nippon Medical & Chemical Instruments Co. Ltd.), and seed setting rates were determined, following the previously described method (Toriyama et al. 2024).

### Northern blot analysis of *orf288*

Total RNA was isolated from anthers at the flowering stage; the *orf288* RNA was detected using the *orf288* RNA probe, as described by Toriyama et al. (2024).

### Prediction of PPR-binding sites

A mitochondrial targeting signal peptide was predicted using MitoFates (Fukasawa et al. 2015; https://mitf.cbrc.pj.aist.go.jp/MitoFates/cgi-bin/top.cgi (Accessed Jun 6, 2025)). A two-letter PPR code was extracted using the PPRFinder server (Cheng et al. 2016; Gutmann et al. 2020; https://ppr.plantenergy.uwa.edu.au/ (Accessed Jun 6, 2025)). Based on the probability score reported by Kobayashi et al. (2019), the base preferences and PPR-binding sites were predicted, as described by Takatsuka et al. (2025).

## Results

### *Rf*-like PPR genes of T65 as candidates of *orf288* regulators

Previously, we reported that the *Rf* genes of the *O. glaberrima* CMS lines with NIP or T65 cytoplasm are linked to the SSRH10045 locus, corresponding to IRGSP-1.0 position (19.01 Mb in chromosome 10). Based on RAP-DB, four *Rf*-like PPR genes are highly expressed in the anthers of NIP; these PPR genes (with distinct locus ID in RAP-DB) have been named based on the number of encoded amino acids: *PPR461* (Os10g0495100), *PPR782*/*rf4* (Os10g0495200), *PPR794* (Os10g0497300), and *PPR506*/*rf1b* (Os10g0499500) (Supplementary Figure S1). To explore the *Rf*-like PPR genes in the T65 genome, we determined the whole-genome sequence. The nucleotide sequences of the *Rf*-like PPR genes in T65 were identical to those in NIP; however, *PPR782*/*rf4* contained an A1620T synonymous mutation. No additional *Rf*-like PPR genes were detected in the T65 genome (Supplementary Figure S1). *PPR506*/*rf1b* was excluded from the *Rf* candidate*s* because its functional allele is involved in the post-transcriptional degradation of the *orf79*-containing transcripts responsible for BT-CMS, which shows no sequence similarity to *orf288* (Wang et al. 2006).

We also determined the nucleotide sequences of *O. glaberrima*, a maintainer line for the CMS line with T65 cytoplasm (TGA), considering that a PPR gene, functional in NIP but non-functional in *O. glaberrima*, is a potential *Rf*-gene regulating the *orf288* expression. Each PPR gene, corresponding to *PPR461*, *PPR782*/*rf4*, or *PPR794*, was amplified through PCR using the primers listed in Supplementary Table S1. All three *Rf*-like PPR genes of *O. glaberrima* were non-functional: A deletion of 21 nucleotides was found in the first PPR motif in the *PPR461*; a 1338 bp-long 5′ half side is 97% identical to that of NIP but completely distinct thereafter in *PPR782*/*rf4*; a one-bp insertion in the 11th nucleotide led to a frame shift in *PPR794* (Supplementary Table S1, Supplementary Figure S1). Hence, all three *Rf*-like PPR genes of T65, *PPR461*, *PPR782*/*rf4*, and *PPR794*, were candidate *Rf* genes for TGA. We expected that the CRISPR-Cas9-mediated knockout of these PPR genes in T65 would allow the expression of *orf288* and cause CMS in T65, in the same manner as TGA.

### Expression of *orf288* and seed settings in mutants of *Rf*-like PPR genes

**Figure 2:**
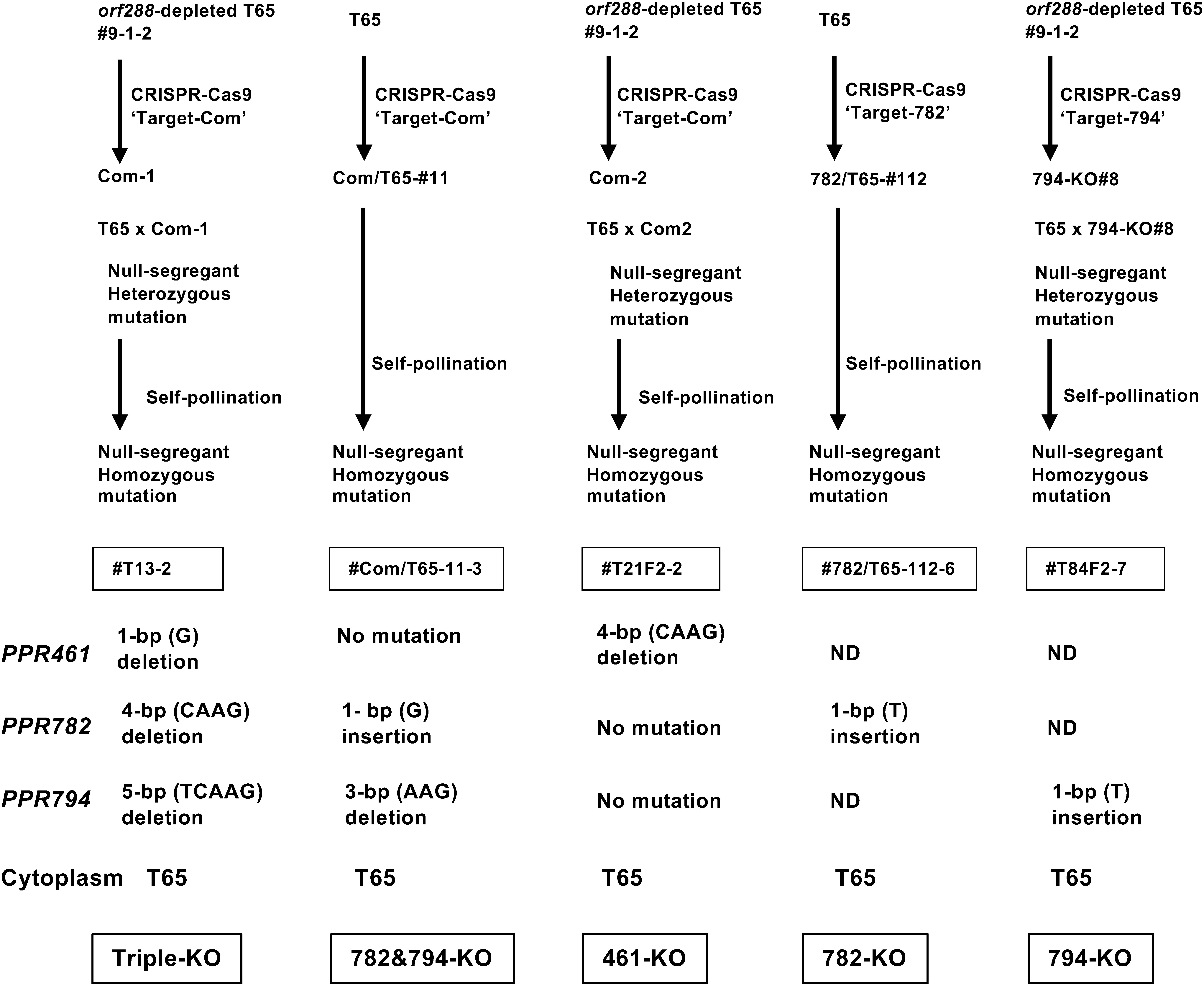
Figure 2. Pedigree of PPR-KO plants and mutations in each PPR gene.

To induce CRISPR-Cas9-mediated mutations in *PPR461*, *PPR782*/*rf4* and *PPR794*, a guide RNA was designed based on the common sequence (Target-Com) of *PPR461*, *PPR782*/*rf4* and *PPR794* as well as the sequences specific to individual PPR genes (Target-461, Target-782, and Target-794) (Figure 1). The CRISPR-Cas9 vector was introduced into T65 cells. We obtained several PPR-knockout (KO) plants exhibiting lower seed setting; however, we could not validate that the low seed setting rate was caused by the KO of the PPR genes or by the somaclonal mutations frequently occurring during the transformation process. To confirm the absence of undesirable somaclonal mutations affecting pollen and/or seed fertility, the CRISPR-Cas9 vector was introduced into *orf288*-depleted T65 (Toriyama et al. 2024), and the high seed setting rate in the resulting PPR-KO plants was confirmed; subsequently, their pollen was used for a crossing with the T65 (pistil), to generate PPR-KO plants with T65 mitochondria carrying *orf288*. The pedigree of the PPR-KO plants is shown in Figure 2. The nucleotide sequences in and around the target sites were determined using the primers listed in Supplementary Table S2. The chromatograms of each nucleotide sequence are shown in Supplementary Figure S2. We selected null segregants lacking the hygromycin phosphotransferase (*HPT*) gene and confirmed the presence of *orf288* in them (Supplementary Figure S3). Finally, the following null-segregant plants with homozygous mutations were selected: *PPR461-*KO plants carrying a deletion of CAAG, *PPR782-*KO plants carrying an insertion of T, *PPR794-*KO plants carrying an insertion of T, double KO plants carrying an insertion of G in *PPR782* and a deletion of AAG in *PPR794*, and triple-KO plants carrying a deletion of G, CAAG, and TCAAG in *PPR461*, *PPR782*, and *PPR794*, respectively.

**Figure 3:**
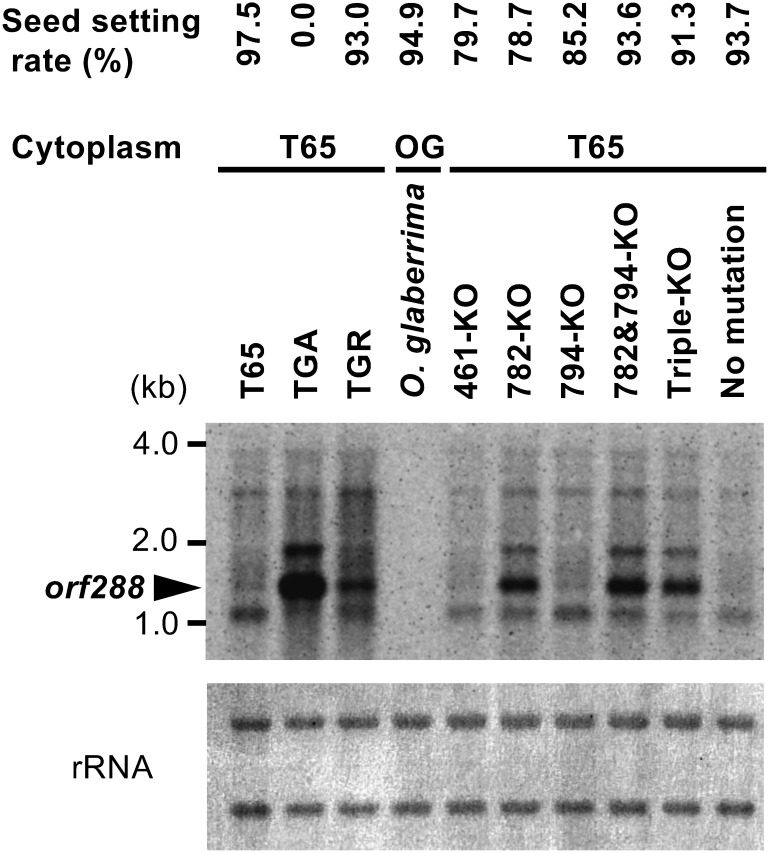
Figure 3. Northern blot analysis of anthers of different rice lines at the flowering stage, conducted to detect *orf288* RNA in PPR-KO plants. *Oryza glaberrima* CMS line (TGA) and restorer line (TGR) were used as controls. All the PPR-KO plants, TGA, TGR have T65 mitochondria carrying *orf288* (Supplementary Figure S3). Seed setting rate is presented above the name of each plant. Knockout of *PPR782* allowed the expression of *orf288*, which was insufficient to induce male sterility.

Total RNA was isolated from the anthers of these PPR-KO plants at the flowering stage and used for northern blot analysis of *orf288.* The *orf288* RNA was not detected in the *PPR461*-KO or *PPR794*-KO plants in the same manner as in T65 (Figure 3). Contrastingly, it was detected in the *PPR782*-KO, *PPR782&PPR794*-double KO, and *PPR461&PPR782&PPR794*-triple KO plants; however, the levels of *orf288* RNA detected in these lines were lower than that in TGA (Figure 3).

The seed setting rates of these PPR-KO plants were 78.7–93.6%, indicating high seed setting rates comparable to those of TGR and *O. glaberrima* (Figure 3; Supplementary Table S3). Sterile plants were not detected even in the triple-KO plants, which showed a seed-setting rate of 91.3%.

## Discussion

Although the CMS plants have not yet been generated, our results demonstrate that the T65 allele of *PPR782*/*rf4* contributes to reducing the *orf288* RNA level. The *PPR782*/*rf4* allele in NIP and T65 did not induce the downregulation of *WA352* RNA and restoration of fertility in WA-CMS; NIP and T65 serve as maintainer lines for WA-CMS (Kazama and Toriyama 2014; Zhang et al. 2022; Zhao et al. 2023). In ‘IR 24’, the functional *Rf4* allele encodes a protein that triggers the degradation of *WA352*-containing RNA; however, associated cleavage is inconspicuous. Neither of the *PPR782*/*Rf4* alleles encodes a Restorer-of-fertility C-terminal domain, which is associated with cleaving RNA targets (Huynh et al. 2023). *PPR782*/*rf4* could be involved in the degradation of the *orf288* transcripts rather than the suppression of the *orf288* transcription.

PPR proteins bind to specific sequences of RNA molecules, and the sequences recognized by PPR proteins can be predicted based on two-letter PPR code, the combination of amino acid residues present at the 5th and 35th within each repeat (Cheng et al. 2016). PPR782a/Rf4-IR 24 and PPR782/rf4-T65 exhibited 95% amino acid identity and harbored 18 PPR motifs (Supplementary Figure S4). Five of 18 PPR codes differed between PPR782a/Rf4-IR 24 and PPR782/rf4-T65, leading to differences in base preference (Supplementary Figure S5). The predicted RNA binding site of the PPR782a/Rf4-IR 24 in the coding sequence (CDS) of the *WA352* RNA is 5′-GGUUGCACCAAAUGCUCG-3′ (*p*-value=0.0000252) (Takatsuka et al. 2025), while the binding site with *p*-value <0.001 was not predicted for the PPR782/rf4-T65 in the CDS of the *orf288* RNA. The differences in PPR codes at the PPR6, PPR7, PPR12, PPR13, and PPR14 motifs potentially affected their binding ability to WA352 RNA in PPR782/rf4-T65 (Supplementary Figure S5), which is supported by a previous finding reflecting the crucial association of amino acid substitutions in the PPR13, PPR14, and PPR15 motifs with fertility restoration in WA-CMS (Zhao et al. 2023). Further research to explore the binding site of PPR782/rf4-T65 and the affinity of each PPR protein for *WA352* and *orf288* RNAs can elucidate the mechanisms underlying the effectiveness of PPR782/rf4-T65 in the reduction of *orf288* RNA.

Although the knockout of *PPR782*/*rf4* allows for the accumulation of *orf288* RNA, the level of *orf288* RNA, lower than that in TGA, was insufficient for the CMS induction (Figure 3). In *O. glaberrima*, only two *Rf*-like PPR genes were reported on chromosome 2; moreover, no *Rf*-like PPR genes have been reported on chromosome 10 (Melonek et al. 2016). *O. glaberrima*, with no *orf288* in its mitochondrial genome, does not require *Rf*-like PPR genes to suppress *orf288* expression (Toriyama et al. 2024). Contrastingly, T65 would carry an additional PPR gene other than *PPR782*/*rf4*, which contributes to reinforcing the degradation of the *orf288* RNA.

Such an additional gene might be a non-PPR gene, as reported for *Rf2* encoding a glycine-rich protein in Lead Rice-type CMS, which reduces the accumulation of the *orf79*-containing RNA (Itabashi et al. 2011). *Rf5* and *Rf6*-encoded PPR proteins in HL-CMS were reported to act as a restoration of fertility complex (RFC; 400–500 kDa) comprising the following components: a glycine-rich protein containing the RNA recognition motif (GRP162), a DUF1620-containing and WD40-like repeat protein, mitochondrially localized hexokinase 6 (OsHXK6), and an unknown factor exhibiting endoribonuclease activity (Hu et al. 2012; Huang et al. 2015; Qin et al. 2016). A certain component of RFC may influence the processing of *orf288*-containing RNA through PPR782/rf4-T65. The genes encoding detoxification-related enzymes, such as mitochondrial aldehyde dehydrogenase in maize (Liu et al. 2001) and peptidase-like protein in sugar beet (Kitazaki et al. 2015), might be involved in fertility restoration of the *orf288*-mediated CMS. Efforts are currently in progress to identify such an additional gene.

The CRISPR-Cas9-mediated knockout of such an additional gene, as well as *PPR782*/*rf4*, would allow the accumulation of *orf288* RNA, as in TGA, and establish a CMS line of T65. Establishment of *orf288*-mediated CMS lines of other *japonica* rice varieties would also be feasible, as all previously investigated *japonica* cultivars carry *orf288* in their mitochondrial genome (Toriyama et al. 2024).

## Abbreviations

BT-CMS: Boro-Taichung 65 type CMS
CDS: coding sequence
CMS: cytoplasmic male sterility
HL-CMS: Hong-Lian type CMS
HPT: hygromycin phosphotransferase
KO: knockout
NIP: Nipponbare
PPR: pentatricopeptide repeat protein
RAP-DB: Rice Annotation Project Database
Rf: restorer of fertility
RFC: restoration of fertility complex
T65: Taichung 65
TGA: *Oryza glaberrima* CMS line with T65 cytoplasm
TGR: *Oryza glaberrima* restorer line with T65 cytoplasm
WA-CMS: wild abortive type CMS

## Accession numbers

Nucleotide sequence data were deposited to DDBJ under the accession No. LC882417. Next-generation sequencing data used in this study is available in the DDBJ Sequence Read Archive under accession number PRJDB35732.

## Description of supplementary files

Supplementary Table S1. Primers used for the sequencing of *O. glaberrima* PPR genes.

Supplementary Table S2. Primers used for determining sequences in and around target sites.

Supplementary Table S3. Summary of seed setting rates in PPR-KO plants.

Supplementary Figure S1. PPR-containing genes in Taichung 65 (T65) and locus ID of Nipponbare in RAP-DB.

Supplementary Figure S2. Chromatograms reflecting the nucleotide sequences of *PPR461*, *PPR782*, and *PPR794* genes in PPR-KO plants.

Supplementary Figure S3. The selection of null-segregants lacking hygromycin phosphotransferase (HPT) gene and confirmation of the presence of *orf288*.

Supplementary Figure S4. Amino acid sequences of PPR782/rf4-T65 and PPR782a/Rf4-IR 24.

Supplementary Figure S5. Base preference of PPR782/rf4-T65 and PPR782a/Rf4-IR 24 predicted based on amino acid residues for the two-letter PPR code in each PPR motif.
