# Supplementary material for: Non-functional allele of RESTORER OF FERTILITY 4 is functional for the reduction of orf288 RNA in japonica rice
Source: Plant Biotechnol (Tokyo). 2026 Mar 25;43(1):83–8. doi: 10.5511/plantbiotechnology.25.1116a (PMC13170784; doi:10.5511/plantbiotechnology.25.1116a)
Supplement: Supplementary Data [file plantbiotechnology-43-1-25.1116a-s001.pdf]

Supplementary information

Non-functional allele of *RESTORER OF FERTILITY 4* is functional for the reduction of *orf288* RNA in *japonica* rice

Kinya Toriyama[1*], Yuko Iwai[1], Shinya Takeda[1], Ayumu Takatsuka[2], Keisuke Igarashi[1], Tomohiko Kazama[2]

1 Graduate School of Agricultural Science, Tohoku University, Sendai, Miyagi, 980-8572, Japan
2 Graduate School of Bioresource and Bioenvironmental Science, Kyushu University, Fukuoka, Fukuoka 819-0395, Japan.

*Corresponding author

Supplementary Table S1. Primers used for the sequencing of *O. glaberrima* PPR genes

	Amplification of template		Direct sequencing	
	Name	Primer sequence	Name	Primer sequence
PPR461	Tori-24F	AAGGTACCACCTGTCAGCTAGGGCGGGC	Tori024F	AAGGTACCACCTGTCAGCTAGGGCGGGC
			1-6	TACAGCGTTCTCATCGGTTG
			1-5-1	TTGAGAAGAATGGTGCAGGA
			1-7	GGGCAGTTGACAGAGGCTAT
	Og-PPR461-R2	CACGTGCTAAGTTCAGTCTC	1-8	GCATCTGTCCAGACACCATT
			1-9	AGCATAGCCGATCAGATGGA
			1-10	CAACCCGAAAGCAAGAACTC
			1-11	TGGGGTGGAACCTAATTGTG

PPR782	Tori-25F	AAGGTACCTGGCGCCCGCGGGGGCAGGC	Tori018R	TTCAAGCCGTAGATCGAGGC
			Tori020R	AGGGCTCCTTTGGTAGCATA
			Tori025F	AAGGTACCTGGCGCCCGCGGGGGCAGGC
			Tori030F	TATTGCTCTTCAGGGCAGCC
	2-14R	AATAGCATGCACCTCCTGAC	2-14R	AATAGCATGCACCTCCTGAC
			4-16	CTTTGCAAGTCAGGCAGTGT
			PPR517_subF1	GGGAAACCAGATGCTGTAGG

PPR794	Tori-11F	CACCGGATCCAGTCCGCTAGGGCGGGCGGGC	Tori011F	CACCGGATCCAGTCCGCTAGGGCGGGCGGGC
			Tori011R	AAAGGATCCATTTCAAAGCTGCAAAAGGCT
			Tori015F	TGCAAAGAAGGGAGGGTTAT
			Tori017F	ATAACCAGGGCTGGCACTTA
	Tori11R	AAAGGATCCATTTCAAAGCTGCAAAAGGCT	Tori018R	TTCAAGCCGTAGATCGAGGC
			Tori020R	AGGGCTCCTTTGGTAGCATA
			2-11	CATAGTGCTCCGCAGAATGA
			2-12	CCTGCTTCAGGGGTATGCTA

Underline indicates the added *Kpn* I or *Bam* HI site.

Nucleotide sequence

```
>PPR461
ATGGCGCGCCGCGCCGCTTCCCGCGCTGTTGGCTCGGAGGGCTCGATCCAAGGGCGAGGGGGCCGCGCGGGGGGCAATGGCGCCGGGGACGCACGCCACGTGTTCGACGAATTGCTTCGGCGTGGCAAGGGCGCCACGATCTACGGCTTGAACCGC
GCCCTCGACGACGTCGCGCGTCACAGCCCCGCGGCCGCCGTGTCCCGCTACAACCGCATGGCCCGAGCCGGCGCCGACGAGGTAACTCCCAACTTGTACACCTACAGCGTTCTCATCGGTTGCTGCTGCCGGGCGGGCCGCTTGGACAATGTCATT
AAGAAGGGATTTAGAGTGGAAGCCATTACCTTCACTCCTCTGCTCAAGGGCCTCTGTGCCGACAAGAGGACGAGCGACGCAATGGACATAGTGCTCTGCAGAATGACCCAGCTCGGCTGCATACCAAATGTCTTCTCCTGCACCATTCTTCTCAAG
GGTCTGTGTGATGAGAACAGAAGCCAAGAAGCTCTCGAGCTGCTCCAAATGATGCCTGATGATGGAGGTGACTGCCCACCTGATGTGGTGTTGTACAACACCGTCATCAATGGCTTCTTCAAAGAGGGGGATCCGGACAAAGCTTACGCTACATAC
CATGAAATGTTTGACCAGGGGATTTTGCCAGATGTTGTGACTTACAGCTCTATTATCGCTGCCTTATGCAAGGCTCAAGCTATGGACAAGGCCATGGAGGTACTTAACACCATGGTTAAGAATGGTGTCATGCCTAATTGCAGGACATATAATAGT
ATTGTGCACGGATATTGCTCTTCAGGGCAGTTGACAGAGGCTATTGGATTTCTCAAAAAGATGTGCAGTGATGGTGTCGAACCAGATGTTGTTACTTGTAACTTGCTGATGGATTATCTTTGCAAGAACAGAAGATGCACGGAAGCTAGAAAGATT
TTCGATTCTATGACCAAGTGTGGCCTAAAGCCTGATATTACTACCTATTGTACCCTGCTTCAGGGGTATGCTACCAAAGGAGCCCTTGTTGAGATGCATGATCTCCTGGATTTGATGGTATGGAACGGTATCCAACCTAATCATCATGTATTCAAC
ATTCTAATATGTGCATACGCTAAACAAGAAAAAGTAGATGAGGCAATGCTTGTATTCAGCAAAATGAGGCAGCAAGGATTGAGTCCGAATGCAGTGAACTACAGAACAGTCATAGATGTACTCTGCAAGCTAGGCAGAGTATACGATGCAGTGCTT
ACCTTAAAGCAGATGATCAATGAAGGACTAACCCCTGACATCATTGTATATACCCCCCTAATTCATGGTTTTTGTACCTGTGACAAATGGAAGAAGGCTGAGGAGTTAATTTTTGAAATGTTGGATCAAGGCATCTGTCCAGACACCATTTTCTTT
AGTAGAATAATTTATAATTTTTGCAAAGAAGGGAGAGTTATAGAATCTGAAAAACTCTTTGACTTGATGGCACATACTGGTGTTAAGCCTGATGTCATTACATACAATGCACTCATCAGTGGATATTGCCTAGCCGGTGTTGACGTAAAACACGAG
GCCTGGGAGATCTGCTTAACTCCAGTGCAGGTCCAAAACTCGCCTTCGGGTATGCTTAGCGTGCCAGTTGATTTGATCCTGCAATCAACAAGAAACAAAGACAAAGAAACCGCGGTTAAATCCATAAACGATAGCCGATCGGCTAGGTGCCGATGA
CATATCATTTATCTTTGAGCCGATGTCATATGTGCATCGATCGGCAGTCATTAATAAATAAGAAGGGACTAAATCTATTCGATCGGCTGTAGATATTAACAATATATAGTCCTTATATCGATATATACTTAAATCAAGTGATTGGGATAGATCGGT
CTCCATGCCGAGACAGTATGAATCACTTAGATCGAAATATATATTAATAATGAGATTATATACGTTCATAGCATAGCCGATCAGATGGATCTAGCATGTATCGGTTAATACTCCGATACTACTCTATATTAAGATATTAAAGCAAGTAGAATATAC
TAAACAAAAGCCTAATATACTTAAATGCAGCAATATCTTAATATAAAGGGTAGATTTAACATGTCAATCGAGCATATAGGATAAAAGTAGTTAAATCAGTTAAGATCGGCTGAAACCCCGATACTACCCTAATCGGCAACCAAAGATAGACTAGAG
ATTGAGATTCTAATTACGACTCATAAGATCAAACTCAACTGATGCAGCTATAAGTATGAAAAGAAGGATAATATCTAGACAATCAAGCCGTTGGATATGTCATAGAGTGGTGGATATCCTATATAATCTAAGTCAACGTCGGTATTTAACCTAATC
GGCTGCCTTCTGGTAACAGACGTTAGCCGATTAGAGGTTAGATAACGATATTGCCAAAGATTATGTAAGATATATGATAACTCGACGAATTATATAAACAAGATTAGAGTGTCATAAAGATGGAAGCACTAATCCCGAGAACGCAAGCCGCCATAA
CAAGTTTTACCTCTTGTTGAAGATCGAAACCGATGCAGCTCAACCCGAAAGCAAGAACTCGTCAAAATAAAACTAAAGCAAAAAGGGTGGCGATGCGCTGAGATTGTTATTGAACGTGTGTTATCGATTACATAGGGTTCGGGTCTATTTATACCC
GAGAATTACAAGATATGTCCATACCGGACACGACTCTTATCCCTAACAAACTCTAAGATACCATAAGTCTTTGCGGCAGACTTTTGCCCAAGCATATCTCTAAGGAAATTATATAAAATATCATAATTAATAGATACAATTGCCTTCCCAGGACTC
TATCCATGCACGGCAATCATCGTGAAGCATATCAACTCAACCCGATGTTGTACACCAAGTCATATTGTCGGAATCGGCTGTATCGGCTTACCCAGTCTGACTCGGACCCAGCTGATCCTGATCGTAGCCGATTCGGACTCCAGCCGATTTTTGCTC
GGTTCTCGAATTGATCTCCGCCTCCGTCTCCACTTCAACCTAATCCCCTTTTCCGATACTGATATTACCAAATTTGGTCGTTAACAGCCGGTAAGATGGATGAAGCAATGAAATTACTCCCTGGCATGGTCTCAGTTGGGGTGGAACCTAATTGTG
TTACTTACCACAGTTTGATTAATGGATATTGTAAAGGTAGTAGGATGGAGGACGCGTTGGTTCTTTTCATAGAGATGGTGAGCAATGGTGTTATTCCTGATATTATCACGTACAACACAATTCTGCATGGTTTGTTTCAGACAGGGAGAATAGCTG
CTGCGAAAGAGCTCTACGTCAGGATTGTTAAAAGAGGAATA
```

```
>PPR782
ATGGCGCGCCGCGTCCCTACCCGCCCGCGCGGCGGCGGCGGCGGCGGCGTCCCACGCTCGGAGGGCTCGATCCAAGGGCGAGGAGGCCGCGCGGGGGGCAGTGGCGCCGAGGACGCACGCCACGTGTTCGACGAATTGCTCCGGCGTGGCAGGGGC
GCCTCGATCTACGGCTTGAACCGCGCCCTCGCCGACGTCGCGCGTCACAGCCCCGCGGCCGCCGTGTCCCGCTACAACCGCATGGCCCGAGCCGGCGCCGACGAGGTAACTCCCGACTTGTGCACCTACAGCATTCTCATCGGTTGCTGCTGCCGC
GCGGGCCGCTTGGACCTCGGTTTCGCGGCCTTGGGCAATGTCATTAAGAAGGGATTTAGAGTGGAAGCCATCACCTTCGCTCCTCTGCTCAAGGGCCTCTGTGCCGACAAGAGGACGAGCGACGCAATGGACATAGTGCTCCGCAGAATGACCGAG
CTCAGCTGCATGCCAGATGTTTTCTCCTGCACCATTCTTCTCAAGGGTCTGTGTGATGAGAACAGAAGCCAAGAAGCTCTCGAGCTGCTGCACATGATGGCTGATGATCGAGGAGGAGGTAGCCCACCTGATGTGGTGTCGTATACCACTGTCATC
CATGGCTTCTTCAAAGAGGGGGATTCAGACAAAGCTTACAGTACATACCATGAAATGCTTGATCGGAGGATTTCACCAAATGTTGTGACTTACAGCTCTATTATTGCTGCGTTATGCAAGGCTCAAGCTATGGACAAAGCCATGGAGGTACTTAAC
ACCATGGTTAAGAATGGTGTCATGCCTGATTGCATGACATATAATAGTATTCTGCATGGATATTGCTCTTCAGGGCAGCCAAAAGAGGCTATTGGAACTCTCAAAAAGATGCGCAGTGATGGTGTCGAACCAAATGTTGTTACTTATAGATCACTG
ATGAATTATCTTTGCAAGAATGGAAGATGCACCGAAGCTAGAAAGATTTTCGATTCTATGACCAAGAGGGGCCTAGAGCCTGATATTGCTACCTATCGTACCCTGCTTCAGGGGTATGCTACCAAAGGAGCCCTTGTTGAGATGCATGCTCTCTTG
GATTTGATGGTACGAAATGGTATCCAACCGGATCATCATGTATTCAAAATTCTAATATGTGCATACGCTAAACAAGAGAAAGTAGATCAGGCAATGCTTGTATTCAGCAAAATGAGGCAGCATGGATTGAATCCGAATGTAGTGTGCTATGGAACA
GTTATAGATGTACTTTGCAAGTCAGGCAGTGTAGATGATGCTATGCTTTATTTTGAGCAGATGATCGATGAAGGACTAACCCCTAACATTTTTTCTGCTTATATTGGATTTATAGGTGTTTATATATAAAGGTAGTTCTACAGTTGTACTTCCACT
TTTAGTGTGGAACACCCTATGCCTTTGGTTTAGTGGCACCGGCATTGCAATTGGGGCAGATGAAACTGAAGAAAGCATATGTGTTAAGCATATCCAAATTCTATTTTAAGTACTTTCAGATTAGTGGCTTGCACTTTGAAGACACCTTCTTTTTTG
GAATAACGAACATGTATTAACTTGACTTTTGGACAGTCTTGTTCTAAAAATAATTGATACTTTCTGTTTCCTGTTAGTGATGGCTATAACCATTAGATGGCTTTTTACAAATTTAGATGATAATTATCATTGTTCAATTTATTGCTGAAATTCTTA
TCTGTATGCATCATTGTTAACACAGCGACAACCAGGAAATTAGTTCTTCTTGAGCTAATCATTTCCCTCCGTAGTTCTGTCATACCTGTGCTGCTATATTACTGTCAGCTGCTTATGATGGCTGCAAAACTCTTTCTTTTGTAGGATCCTGAGTTC
TACAAGTATTTGGAGAAGTGTGATAAAGAGCTCTTGGAATTTGACGATGATGACTTTGAAGTAACACAAAACTATTTAATTATTTATTTTCATTTTTCTGGTGTCTTCCCTGCTGCTTGATATGCTGGAAATGGAAGAACTTGGAGGGAAACCAGA
TGCTGTAGGCAAAGCTGTGAATCTGTTCAGTGTTAAACAGGTATATTTTCTCGTAATACAACACTTCCTTTGGGAGCACTACTTTGAAAAACCTTTTAATAGGTACTTTTTTCTGGCAGGTAGATAAGAAAACAGTGAAGACGCGTGCCTTTCAGG
AAGCATGCATCTTTTCCGCGGTTGATGAGTTGGGCTAAGTATTTTGCCCAATGGAGCTGCTCCATTGCCTTCTTTGAGATGTCCTTTTTAACACTTGTCTGGCTTCAAAACTTTTGCAAAACCAACCGTTAAGGCTGACAGATTCCGGAGAGAGAT
CAAAGATCTTATCCATCAGGTTTGTGCCTGTTTTTTATTCTCTTTTCCAAGAGGTTTTCCACCGGTTTTGGTGTAAATGTTAGCATGTTTCTCGGTGTAAATTGATTCCCACGCATTGCTATATAATCTTGGCCAATGGTTTTTAGTGCCTGTTTT
CAAATTTGGTTAGCTGTCA
```

```
>PPR794
ATGGCGCGCCGGCGCCGCTTCCCGCGCCCGCGCCGGCGCTGTTGGCGCCCTTCGCTCGGAGGGCTCGACCCAAGGGCGAGGGGGCCGCGCGGGGGGCAGTGGCGCCGAGGACGCGCGCCACGTGTTCGACGAATTGCTCCGGCGTGGCAGGGGCGC
CTCGATCTACGGCTTGAACCGCGCCCTCGCCGACGTCGCACGTCACAGCCCCGCGGCCGCCGTGTCCCGCTACAACCGCATGGCCCGAGCCTGCGCCGACGACGTAACTCCCGACTTGTGCACCTACAACATTCTCATCGGTTGCTGCTGCCGCAC
GGGCCGCTTGGACCTCGGTTTCGCGGCCTTGGGCAATGTCATCAAGAAGGGATTTAGAGTGGAAGCCATCAGCTTCACTCCTCTGCTCAAGGGCCTCTGTGCCGACAAGAGGACGAGCGACGCAATGGACATAGTGCTCCGCAGAATGACCGAGCT
CGGCTGCATACCAGATGTCTTCTCCTACAATATTCTTCTCAAGGGTCTGTGTGATGAGAACAGAAGCCAAGAAGCTCTCGAGTTGCTGCACATGATGGCTGATGATCGAGGAGGAGGTAGCCCACCTGATGTGGTGTCGTATACCACTGTCCTCAA
TGGCTTCTTCAAAGAGGGGGATCTGGACAAAGCTTACAGTACATACAATGAAATGCTTGACCAGAGGATTCGCCAAATGTTGTGACCTACAGCTCTATTATTGCTGCGCTATGCAAGGCTCAAACTGTGGACAAGGCCATGGAGGTACTTAACACC
ATGGTTAAGAATGGTGTCATGCCTGATTGCAGGACATATACTAGTATCATGCATGGATATTGCTCTTCAGGGCAGCCGAAAGAGGCTATTGGATTTCTCAAAAAGATGCAGAGTGATGGTGTCGAACCAGATGTTGTTACTTATAACTCGCTCATG
GATTATCTTTGCAAGAACGGAAGATGCATGGAAGCTAGAAAGATTTTTGATTCTATGACCAAGAGGGGCCTTAAGCCTGATATTACTACCTATGGTACCCTGCTTCAGGGGTATGCTACCAAAGGAGCCCTTTTTGACATGCATGCTCTCTTGGAT
TTGATGGTACGAAACGGCATCCACCCTGATCATTATGTTTACAACATTCTAATATGTGCATACGCTAAACAAGAGAAAGTAGAAGAGGCAATGCTTGTATTCAGCAAAATGAGGCAGCAAGGATTGAATCCGAATGCAGTGACCTATGGAACAGTT
ATAGGCATACTTTGCAAGTCAGGCAGAGTAGAAGATGCTATGCGTTATTTTGAGCAGATGATCGATGAAAGACTAAGCCCTGGCAACATTGTTTATAACTCCCTAATTCATAGTCTCTGTATCTTTGACAAATGGGACAAGGCTAAAGAGTTAATT
CTTGAAATGTTGGATCGAGGCATCTGTCTGGACACTATTTTCTTTAATTCAATAATTGACAGTCATTGCAAAGAAGGGAGGGTTATAGAATCTGAAAAACTCTTTGACCTGATGGTACGTATTGGTGTGAAGCTCGATATCATTACGTACAGTACT
CTCATCGATGGATATTGCTTGGCAGGTAAGATGGATGAAGCAACGAAGTTACTTGCCAGCATGGACTCAGTTGGAATGAAACCTGATTGTGTTACATATAGTACTTTGATTAATGGCTACTGTAAAATTAGCAGGATGAAAGATGCGTTAGTTCTT
TTTAGGGAGATGGAGAGCAGTGGTGGTAGTCCTGATATTATTACGTATAATATAATTCTGCAAGGTTTATTTCAAACCAGAAGAACTGCTGCTGCAAAAGAACTCTATGTCGGGATTACCAAAAGTGGAACGCAGCTTGAACTTAGCACATACAAC
ATAATCCTTCATGGACTTTGCAAAAACAAACTCACTGATGATGCACTTCGGATGTTTCAGAACCTATGTTCGATGGATTGGAAACTTGAGCCTAGGACTTTCAACATTATGATTGATGCATTGCTTAAAGTTGGCAGAAATGATGAAGCCAAGGAT
TTGTTTGCTGCTTTCTCGTCTAACGGTTTAGTGCCGAATTATTGGACGTACAGGTTGATGGCTGAAAATATTATAGGACAGGGGTTGCTAGAAGAATTGGATCAACTCTTTCTTTCAATGGAGGACAATGGCTGTACTGTTGACTCTGGCATGCTA
AATTTCATTGTTAGGGAACTGTTGCAGAGAGGTGAGATAACCAGGGCTGGCACTTACCTCTCCATGATTGATGAGAAGCACTTCTCCCTCGAAGCATCCACTGCTTCCTTGTTTATAGATCTTTTGTCTGGGGGAAAATATCAAGAATATCATAGG
TTTCTCCCTGAAAAATACAAGTCCTTTATAGAGTCTTTGAGTTGTTGA
```

Supplementary Table S2. Primers used for determining sequences in and around target sites

Target			Amplification of template		Direct sequencing	
	Target sequence	PAM	Name	Primer sequence	Name	Primer sequence
Common	TTATGCAAGGCTCAAGCTA	TGG	Com		Tori13F	AGCGACGCAATGGACATAGT
PPR461	GTACACCTACAGCGTTCTCAT	CGG	Tori-24F	AAGGTACCACCTGTCAGCTAGGGCGGGC	PPR454-1-5-1	TTGAGAAGAATGGTGCAGGA
			Og-PPR461-R2	CACGTGCTAAGTTCAGTCTC		
PPR782	GTCGTATAACACTGTCCTCAA	TGG	Tori-25F	AAGGTACCTGGCGCCCGCGGGGGCAGGC	Tori13F	AGCGACGCAATGGACATAGT
			2-14R	AATAGCATGCACCTCCTGAC		
PPR794	ATCGGTTCCTGCTGCTGCGC	GGG	Tori-11F	CACCGGATCCAGTCCGCTAGGGCGGGCGGGC	Tori-19R	AGCATTTCATGGTATGTAC
			Tori11R	AAAGGATCCATTTCAAAGCTGCAAAAGGCT		

Underline indicates the added *Kpn* I or *Bam* HI site.

Supplementary Table S3. Summary of seed setting rates in PPR-KO plants

	Panicle 1	Panicle 2	Panicle 3	Panicle 4	Average
T65	97.6	97.4			97.5
TGA	0.0	0.0			0.0
TGR	89.5	85.3	97.3	100.0	93.0
O. glaberrima	92.3	97.6			94.9
461-KO	82.5	76.9			79.7
782-KO	88.0	69.4			78.7
794-KO	85.2				85.2
782&794-KO	93.3	87.5	100.0		93.6
Triple-KO	94.4	88.9	93.8	88.2	91.3
No mutation	96.0	91.9	93.1		93.7

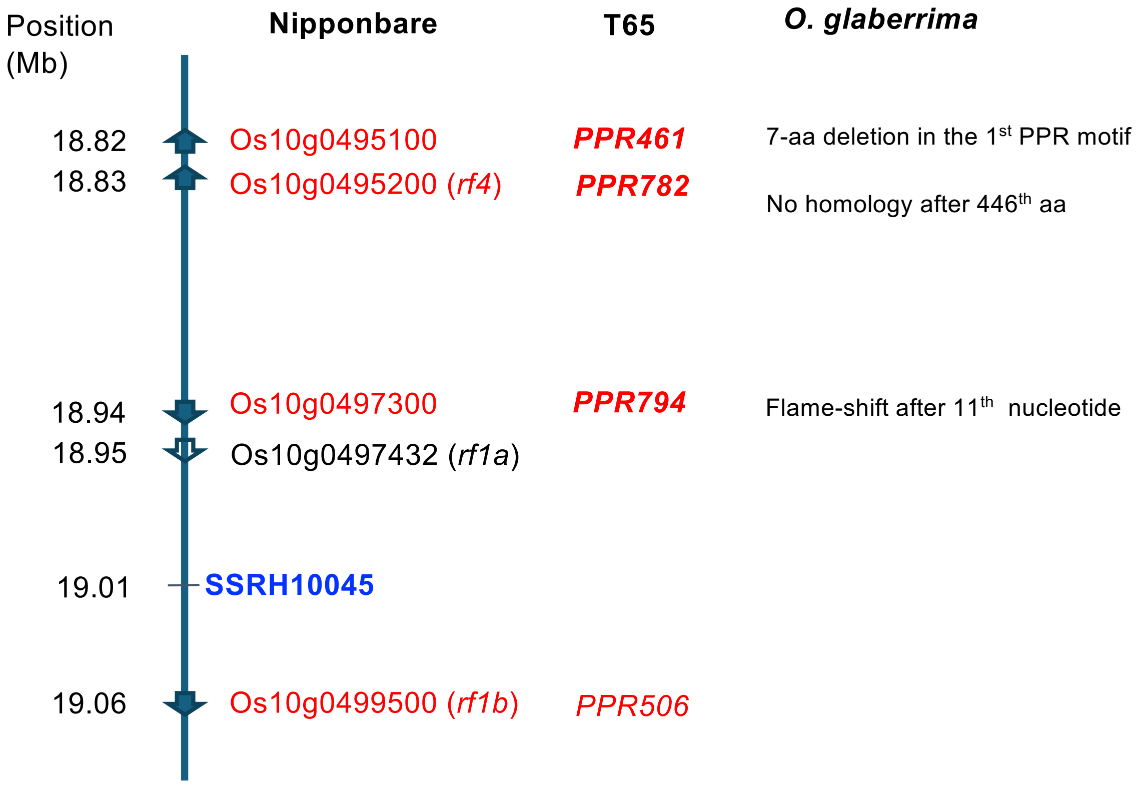

Supplementary Figure S1. PPR-containing genes in Taichung 65 (T65) and locus ID of Nipponbare in RAP-DB. *Rf*-like PPR genes are indicated in red. Features of the *O. glaberrima* amino acid sequence are indicated. Nucleotide sequence data were deposited into DDBJ under the accession No. LC882417.

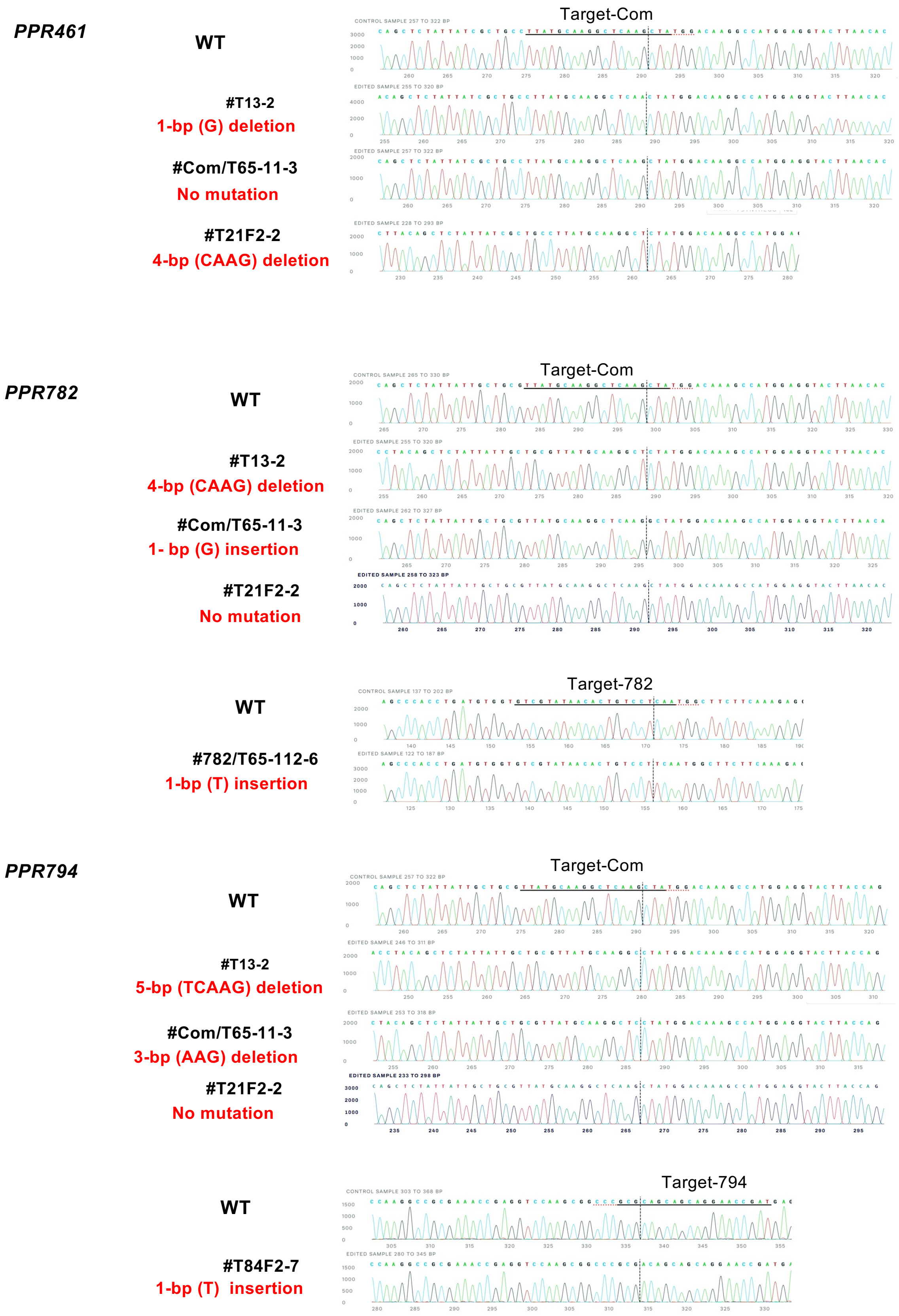

Supplementary Figure S2. Chromatograms reflecting the nucleotide sequences of *PPR461*, *PPR782*, and *PPR794* genes in PPR-KO plants. The target site is underlined.

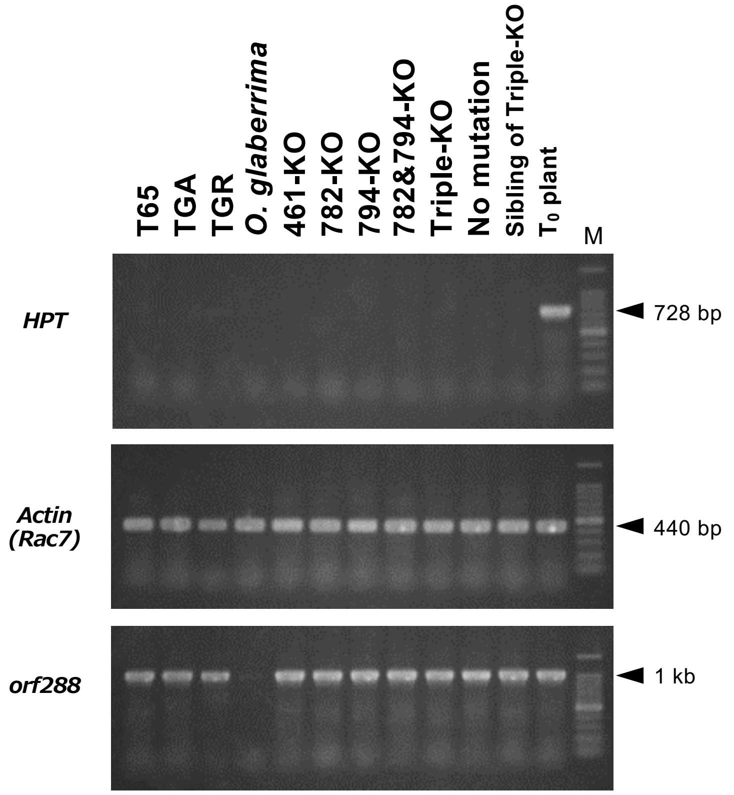

Supplementary Figure S3. The selection of null-segregants lacking hygromycin phosphotransferase (HPT) gene and confirmation of the presence of *orf288*.

```
T65       1   MARRVPTRPRGGGGGGVPRSEGSIQGRGGRAGGSGAEDARHVFDELLRRGRGASIYGLNR   60
              ************************************************************
IR 24     1   MARRVPTRPRGGGGGGVPRSEGSIQGRGGRAGGSGAEDARHVFDELLRRGRGASIYGLNR   60

                                                        PPR 1
T65      61   ALADVARHSPAAAVSRYNRMARAGAGKVTPTVHTYAILIGCCCRAGRLDLGFAALGNVVK   120
              ************************************************************
IR 24    61   ALADVARHSPAAAVSRYNRMARAGAGKVTPTVHTYAILIGCCCRAGRLDLGFAALGNVVK   120

                             PPR 2                         PPR 3
T65     121   KGFRVDAITFTPLLKGLCADKRTSDAMDIVLRRMTELGCIPDVFSYNNLLKGLCDENRSQ   180
              *********************************************** ************
IR 24   121   KGFRVDAITFTPLLKGLCADKRTSDAMDIVLRRMTELGCIPDVFSYNILLKGLCDENRSQ   180

                                   PPR 4                           PPR 5
T65     181   EALELLHMMADDRGGGSPPDVVSYNTVLNGFFKEGDSDKAYSTYHEMLDRGILPDVVTYS   240
              ************************************************************
IR 24   181   EALELLHMMADDRGGGSPPDVVSYNTVLNGFFKEGDSDKAYSTYHEMLDRGILPDVVTYS   240

                                                  PPR 6
T65     241   SIIAALCKAQAMDKAMEVLNTMVKNGVMPDCMTYNSILHGYCSSGQPKEAIGTLKKMRSD   300
              *******************.**************.**.************** *******
IR 24   241   SIIAALCKAQAMDKAMEVLTTMVKNGVMPDCMTYTSIMHGYCSSGQPKEAIGFLKKMRSD   300

                    PPR 7                              PPR 8
T65     301   GVEPNVVTYSSLMNYLCKNGRSTEARKIFDSMTKRGLEPDIATYRTLLQGYATKGALVEM   360
              **.*** **.**************************************************
IR 24   301   GVKPNVFTYRSLMNYLCKNGRSTEARKIFDSMTKRGLEPDIATYRTLLQGYATKGALVEM   360

                           PPR 9                              PPR 10
T65     361   HALLDLMVRNGIQPDHHVFNILICAYAKQEKVDQAMLVFSKMRQHGLNPNVVCYGTVIDV   420
              **************************************************** *******
IR 24   361   HALLDLMVRNGIQPDHHVFNILICAYAKQEKVDQAMLVFSKMRQHGLNPNVVTYGTVIDV   420

                                                  PPR11
T65     421   LCKSGSVDDAMLYFEQMIDEGLTPNIIVYTSLIHGLCTCDKWDKAEELILEMLDRGICLN   480
              **************************************.***.*****...***.*** *
IR 24   421   LCKSGSVDDAMLYFEQMIDEGLTPNIIVYTSLIHGLCTYDKWEKAEELFFKMLDSGICPN   480

                   PPR 12                                   PPR13
T65     481   TIFFNSIIDSHCKEGRVIESEKLFDLMVRIGVKPDIITYNTLIDGCCLAGKMDEATKLLA   540
              *.**.***.. ***********************..************.****** ***.
IR 24   481   TVFFSSIISNLCKEGRVIESEKLFDLMVRIGVKPNVITYNTLIDGCCLGGKMDEAMKLLS   540

                              PPR 14                              PPR15
T65     541   SMVSVGVKPDIVTYGTLINGYCRVSRMDDALALFKEMVSSGVSPNIITYNIILQGLFHTR   600
              .*****.**..***.*******..***.***.***** ******.************.**
IR 24   541   GMVSVGLKPNTVTYSTLINGYCKISRMEDALVLFKEMESSGVSPDIITYNIILQGLFQTR   600

                                   PPR 16                          PPR17
T65     601   RTAAAKELYVSITKSGTQLELSTYNIILHGLCKNNLTDEALRMFQNLCLTDLQLETRTFN   660
              ************************************************************
IR 24   601   RTAAAKELYVSITKSGTQLELSTYNIILHGLCKNNLTDEALRMFQNLCLTDLQLETRTFN   660

                                                  PPR 18
T65     661   IMIGALLKCGRMDEAKDLFAAHSANGLVPDVRTYSLMAENLIEQGSLEELDDLFLSMEEN   720
              *******************************.****************************
IR 24   661   IMIGALLKCGRMDEAKDLFAAHSANGLVPDVWTYSLMAENLIEQGSLEELDDLFLSMEEN   720

T65     721   GCSADSRMLNSIVRKLLQRGDITRAGTYLFMIDEKHFSLEASTASFLLESSPIVWEQISR   780
              ************************************************************
IR 24   721   GCSADSRMLNSIVRKLLQRGDITRAGTYLFMIDEKHFSLEASTASFLLESSPIVWEQISR   780

T65     781   IS   782
              **
IR 24   781   IS   782
```

Supplementary Figure S4. Amino acid sequences of PPR782/rf4-T65 and PPR782a/Rf4-IR 24. Mitochondrial targeting signals are underlined; PPR motifs are highlighted.

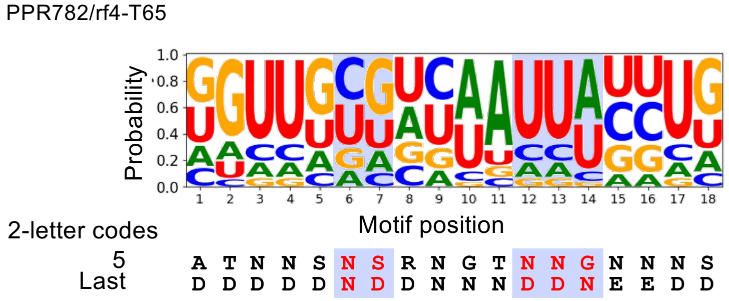

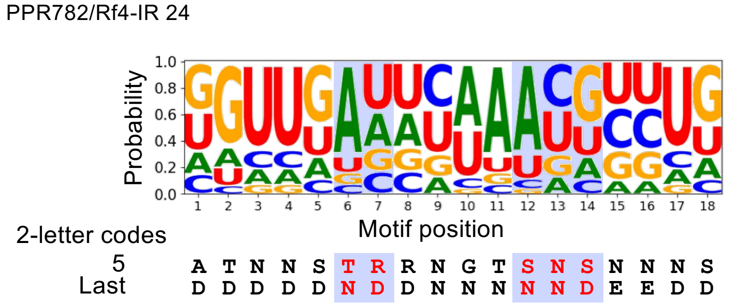

Supplementary Figure S5. Base preference of PPR782/rf4-T65 and PPR782a/Rf4-IR 24 predicted based on amino acid residues for the two-letter PPR code in each PPR motif. Distinct amino acids are indicated in red.
